# Systems Chemical Genetics-Based Drug Discovery: Prioritizing Agents Targeting Multiple/Reliable Disease-Associated Genes as Drug Candidates

**DOI:** 10.3389/fgene.2019.00474

**Published:** 2019-05-29

**Authors:** Yuan Quan, Zhi-Hui Luo, Qing-Yong Yang, Jiang Li, Qiang Zhu, Ye-Mao Liu, Bo-Min Lv, Ze-Jia Cui, Xuan Qin, Yan-Hua Xu, Li-Da Zhu, Hong-Yu Zhang

**Affiliations:** ^1^Hubei Key Laboratory of Agricultural Bioinformatics, College of Informatics, Huazhong Agricultural University, Wuhan, China; ^2^College of Life Sciences and Technology, Huazhong Agricultural University, Wuhan, China; ^3^Sci-meds Biopharmaceutical Co., Ltd., Wuhan, China

**Keywords:** drug discovery, disease associated genes, drug targets, systems chemical genetics, machine learning

## Abstract

Genetic disease genes are considered a promising source of drug targets. Most diseases are caused by more than one pathogenic factor; thus, it is reasonable to consider that chemical agents targeting multiple disease genes are more likely to have desired activities. This is supported by a comprehensive analysis on the relationships between agent activity/druggability and target genetic characteristics. The therapeutic potential of agents increases steadily with increasing number of targeted disease genes, and can be further enhanced by strengthened genetic links between targets and diseases. By using the multi-label classification models for genetics-based drug activity prediction, we provide universal tools for prioritizing drug candidates. All of the documented data and the machine-learning prediction service are available at SCG-Drug (http://zhanglab.hzau.edu.cn/scgdrug).

## Introduction

Finding novel drugs or new uses for old drugs is one of the most important motivations of life sciences. Drug development is a costly process. The rich knowledge accumulated by modern life sciences is, thus, highly expected to reduce the attrition rate during drug development. From a chemical viewpoint, drugs exert therapeutic effects by inhibiting or activating one or more of the target genes/proteins associated with certain diseases. Therefore, gene-disease association information is crucial for drug discovery (Brinkman et al., 2006; Sanseau et al., 2012; Wang Z. Y. et al., 2012; Plenge et al., 2013; Okada et al., 2014; Nelson et al., 2015).

In life sciences, genetics is best dedicated to revealing gene-disease links. Thus, genetics makes great contributions to the pharmaceutical industry. For example, disease-associated genes identified by medical genetics constitute a promising source of drug targets (Brinkman et al., 2006; Sanseau et al., 2012; Wang Z. Y. et al., 2012; Plenge et al., 2013; Okada et al., 2014; Nelson et al., 2015). Moreover, the pathogenesis revealed by genetics is also of high value for drug discovery. If a disease arises from gain of function (GOF) mutation of a target gene, the corresponding drugs must be antagonists or inhibitors; while for a disease induced by loss of function (LOF) mutation of a gene, the targeted drugs must be agonists (Wang and Zhang, 2013).

Thousands of disease-associated genes have been identified by traditional Mendelian genetics and recently developed genome- and phenome-wide association studies (GWAS and PheWAS, respectively). However, nearly all studies attributed diseases to variations at a single genetic locus. Most diseases are caused by multiple pathogenic factors (Yildirim et al., 2007; Hopkins, 2008; Guney et al., 2016); thus, a majority of the identified links between diseases and single genetic variations are not strong enough to have therapeutic value. For example, only ~5% of the drug-disease associations derived from PheWAS are supported by clinical evidence (Rastegar-Mojarad et al., 2015). Thus, to utilize the medical genetic information more efficiently in drug development, we should aim at multiple genes associated with certain diseases rather than a single pathogenic factor to identify potential drugs. To test this hypothesis, we retrieved the genes responsible for various disorders and collected the chemical agents targeting these genes. A comprehensive analysis on the relationships between agent activity/druggability and target genetic characteristics revealed that the agents targeting multiple pathogenic factors were more likely to show desired medicinal activities and to be clinically approved. The therapeutic potential of agents can be enhanced with the consolidation of genetic links between targets and diseases. These observations allowed us to predict agent activities using machine learning methods, which are definitely helpful to prioritize drug candidates.

## Results

### Data Preparation and Validation

**Figure 1:**
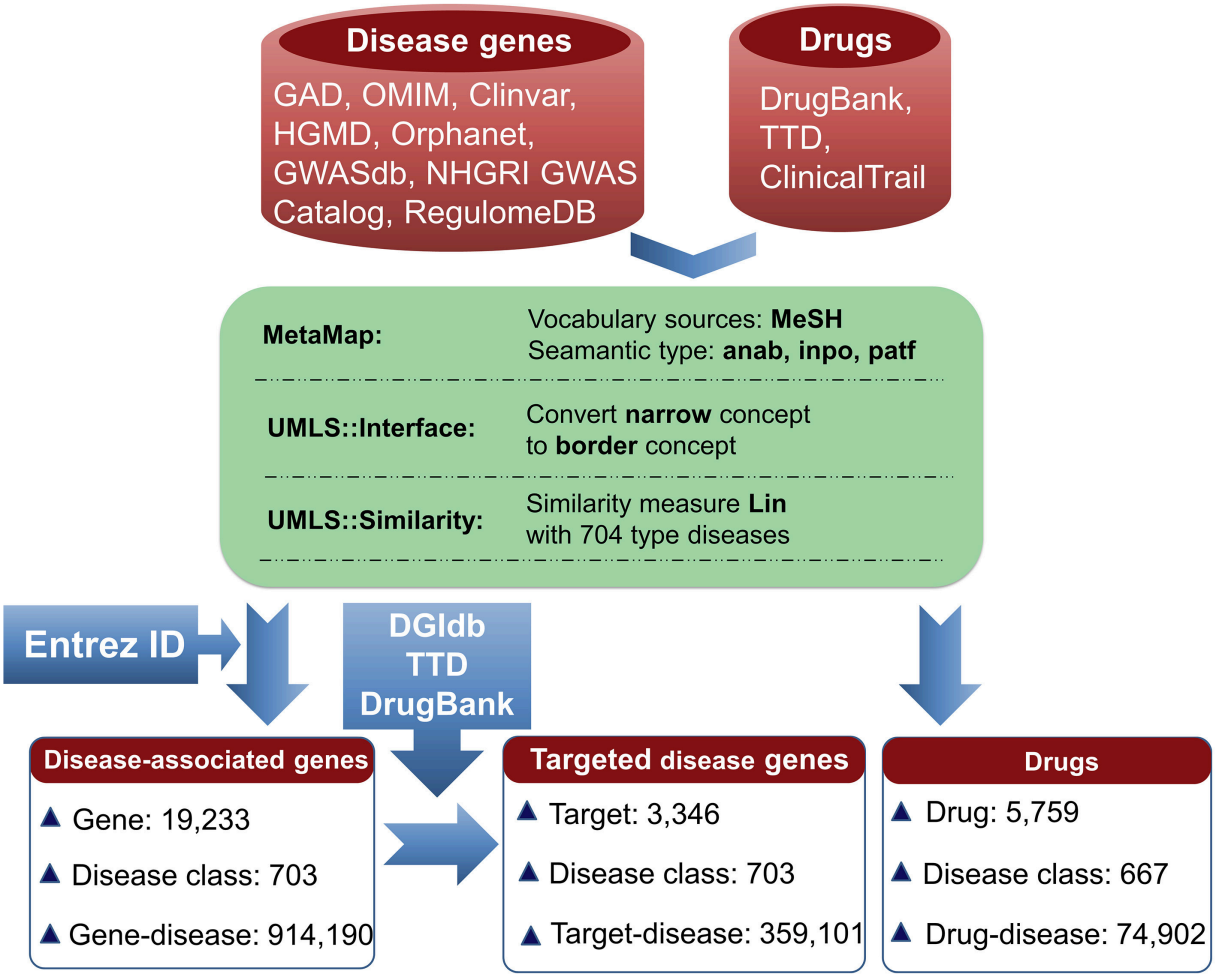
Pipeline for data processing. Disease-associated genes were derived from eight databases. Agent activities were obtained from TTD, DrugBank, and ClinicalTrials. The disease terms of genes and the indication annotations of agents were uniformed to UMLS concepts using MetaMap. Using the disease classes provided by pharmaprojects (Similarity threshold: 0.75), 703 types of diseases for 19,233 genes were identified, resulting in 914,190 gene-disease pairs. Through searching DGIdb, TTD, and DrugBank, 3,346 genes were targeted by 14,558 agents. 3,346 targets were associated with 703 diseases, resulting in 359,101 gene-disease pairs; 5,759 agents were indicated for treating 667 diseases, resulting in 74,902 agent-disease pairs.

The information for agent-target interaction was obtained through retrieving Drug-Gene Interaction database (DGIdb) (Wagner et al., 2015), Therapeutic Target Database (TTD) (Qin et al., 2014), and DrugBank (Law et al., 2014). Only the clinically supported or approved activities of the agents were used in the present study, which were derived from DrugBank, TTD, and ClinicalTrials (Zarin et al., 2011; Law et al., 2014; Qin et al., 2014). The disease-associated gene information was derived from the following eight databases: Genetic Association Database (GAD) (Becker et al., 2004), Online Mendelian Inheritance in Man (OMIM) (Hamosh et al., 2005), Clinvar (Landrum et al., 2014), Orphanet (http://www.orpha.net/consor/cgi-bin/index.php), DisGeNET (Piñero et al., 2015), INtegrated TaRget gEne PredItion (INTREPID) (Chen and Tian, 2016), GWASdb (Nelson et al., 2015), and The Human Gene Mutation Database (HGMD) (Wang X. et al., 2012) (Figure 1).

To facilitate the present analysis, a natural language processing tool MetaMap was used to convert disease terms of genes and indication annotations of agents to Unified Medical Language System (UMLS) concepts (Aronson, 2001), where the Medical Subject Headings (MeSH) thesaurus was selected as the vocabulary source of UMLS (Liu et al., 2014). Using the disease classes provided by pharmaprojects (Similarity threshold: 0.75) (Mcinnes et al., 2009), the chemical agents were indicated for treating 667 disease classes and the disorder-related genes were associated with 703 disease classes (Figure 1). All of the data are freely available at SCG-Drug (http://zhanglab.hzau.edu.cn/scgdrug).

**Figure 2:**
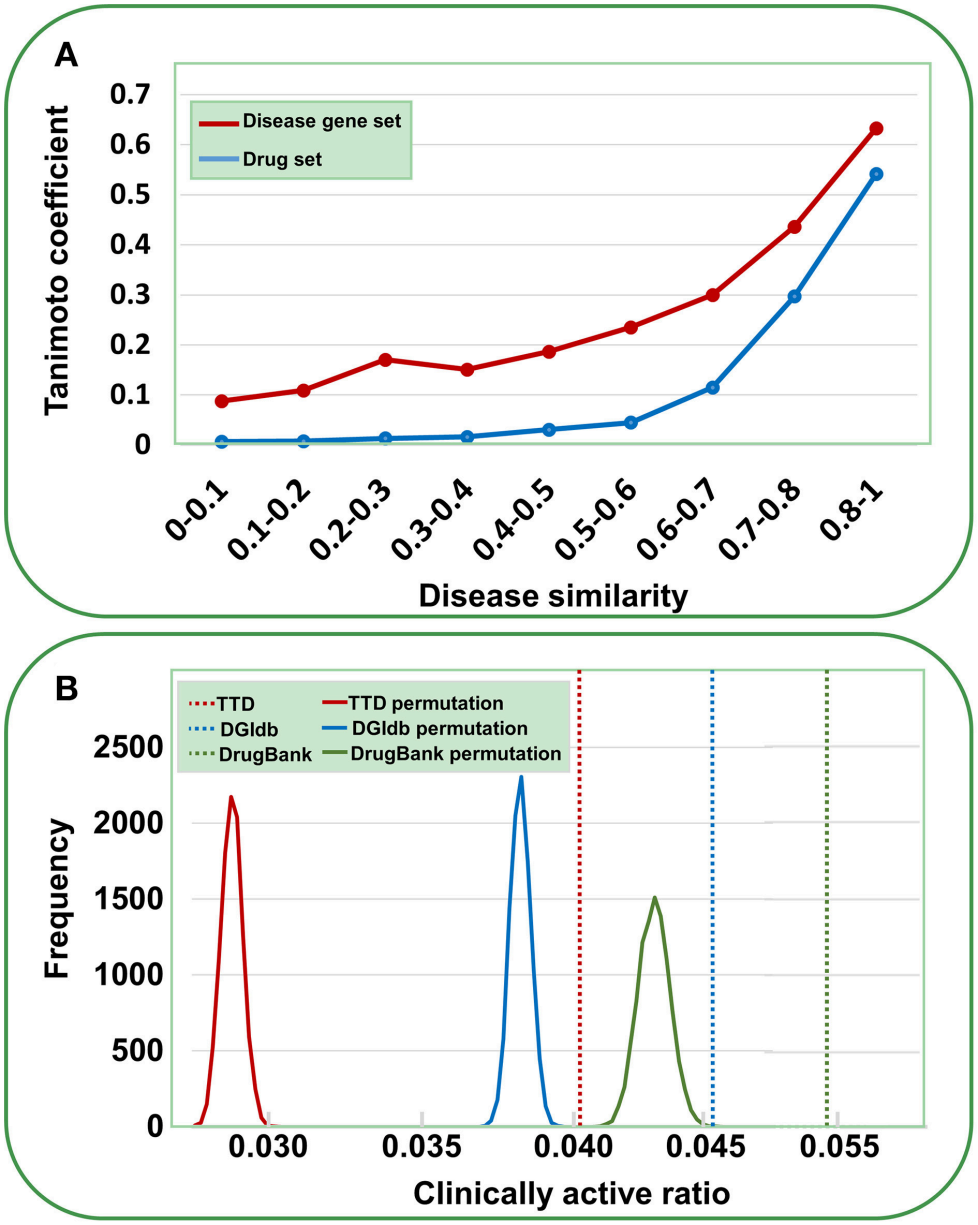
Validation of gene-disease pairs, agent-disease pairs and agent-target pairs. **(A)** Correlations between disease similarity and disease gene set distance or drug set distance. The disease similarity was measured using the UMLS::similarity, and the disease gene set or drug set distance was characterized by Tanimoto coefficient. **(B)** Clinically active ratios of genetics-implicated agent activities. The red, brown, and green vertical dashed lines indicate the clinically active ratios derived from real agent- target pairs in TTD, DGIdb, and DrugBank, respectively. The curves show the clinically active ratio frequency distributions for 10,000 random permutations of agent-target pairs.

Data validation was performed by the following analyses. First, we assessed the reliability of the gene-disease pairs by examining whether similar diseases cover similar gene sets. The disease similarity was measured using UMLS::similarity (Mcinnes et al., 2009); the disease gene set distance was calculated using the Tanimoto coefficient (see Methods). As shown in Figure 2A, a definite correlation exists between disease similarity and gene set distance. That is, if two diseases exhibit similar symptoms, then these diseases tend to involve similar genes, validating the identified gene-disease pairs. Then, we used a similar method to evaluate the quality of agent-disease pairs. A good correlation was observed between disease similarity and agent set distance (Figure 2A), supporting the reliability of agent-disease pairs. Therefore, one can infer the activities of agents through their target-associated genetic diseases, provided the agents and the targets are truly linked. As illustrated in Figure 2B, for the agents in TTD, DGIdb, and DrugBank, 4.1, 4.7, and 5.3% of their genetics-implicated activities are supported by clinical trials, respectively, comparable with the PheWAS-based activity prediction efficiency (Rastegar-Mojarad et al., 2015). However, if the agents were randomly assigned with targets (for 10,000 times), the clinically supported activities derived from genetic predictions are significantly rarer than those from real agent-target pairs (Figure 2B, *P* < 10^−4^). This 10,000-permutation test validates the agent-target associations.

### Dependence of Agent Activity/Druggability on Target Quantity

**Figure 3:**
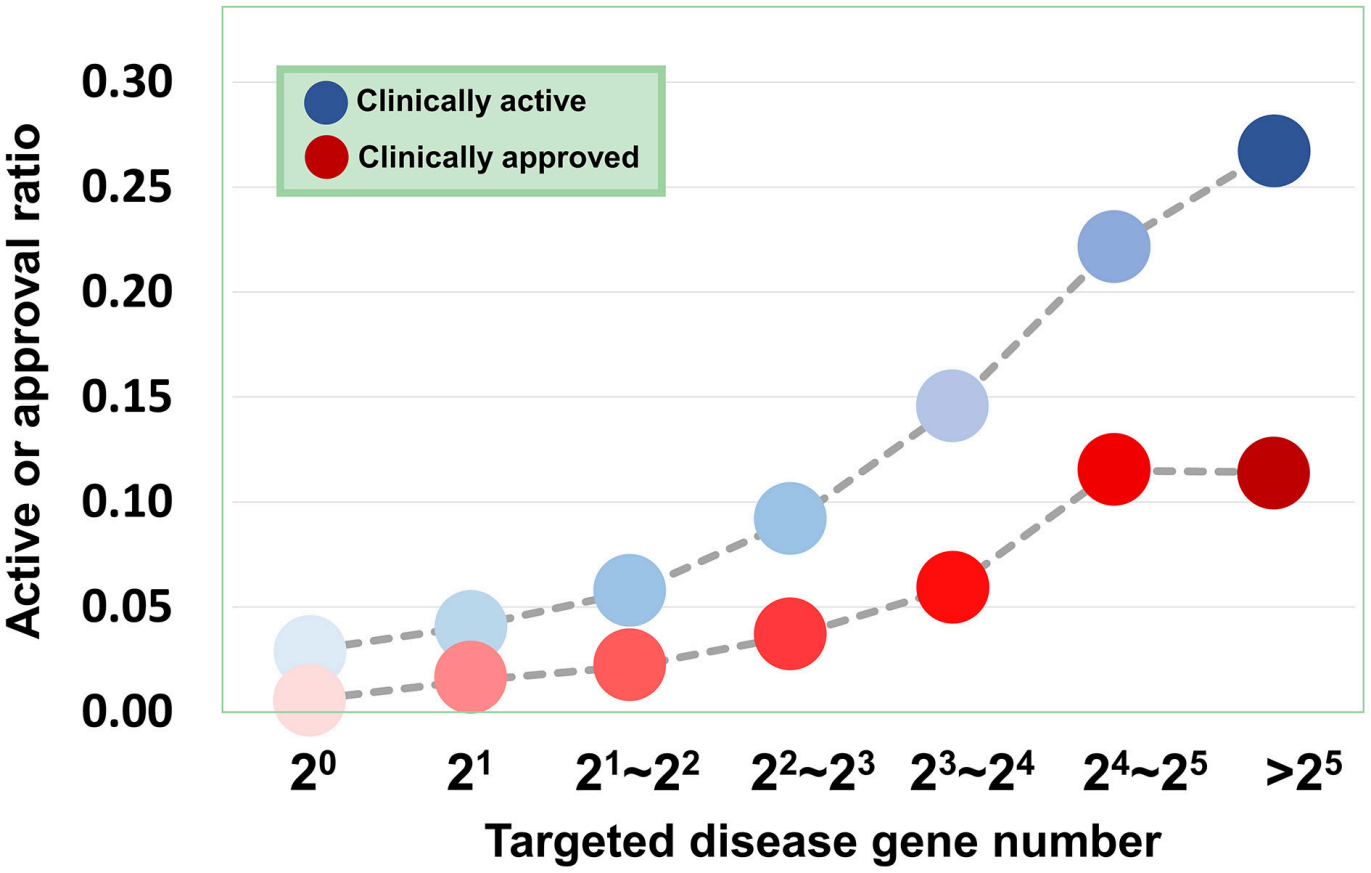
Dependence of agent activity/druggability on target quantity. Therapeutic potential of agents increases with increasing number of targeted disease genes.

Based on the validated data, we can investigate how the agent activity/druggability depends on the target characteristics. As illustrated in Figure 3, for the agents targeting a single disease gene, 3.0% of genetics-derived activities are supported by clinical test and only 0.6% are clinically approved (Table S1). For agents targeting two disease-associated genes, 4.1% of genetics-implicated activities are clinically supported, and 1.5% have been introduced to the market (Table S1). The clinically active ratio of agents culminates to 26.7%, and the approval ratio is up to 11.4%, when the agents targeting tens of disorder genes. Together, the therapeutic potential of agents increases steadily with increasing number of targeted disease genes (Figure 3).

**Figure 4:**
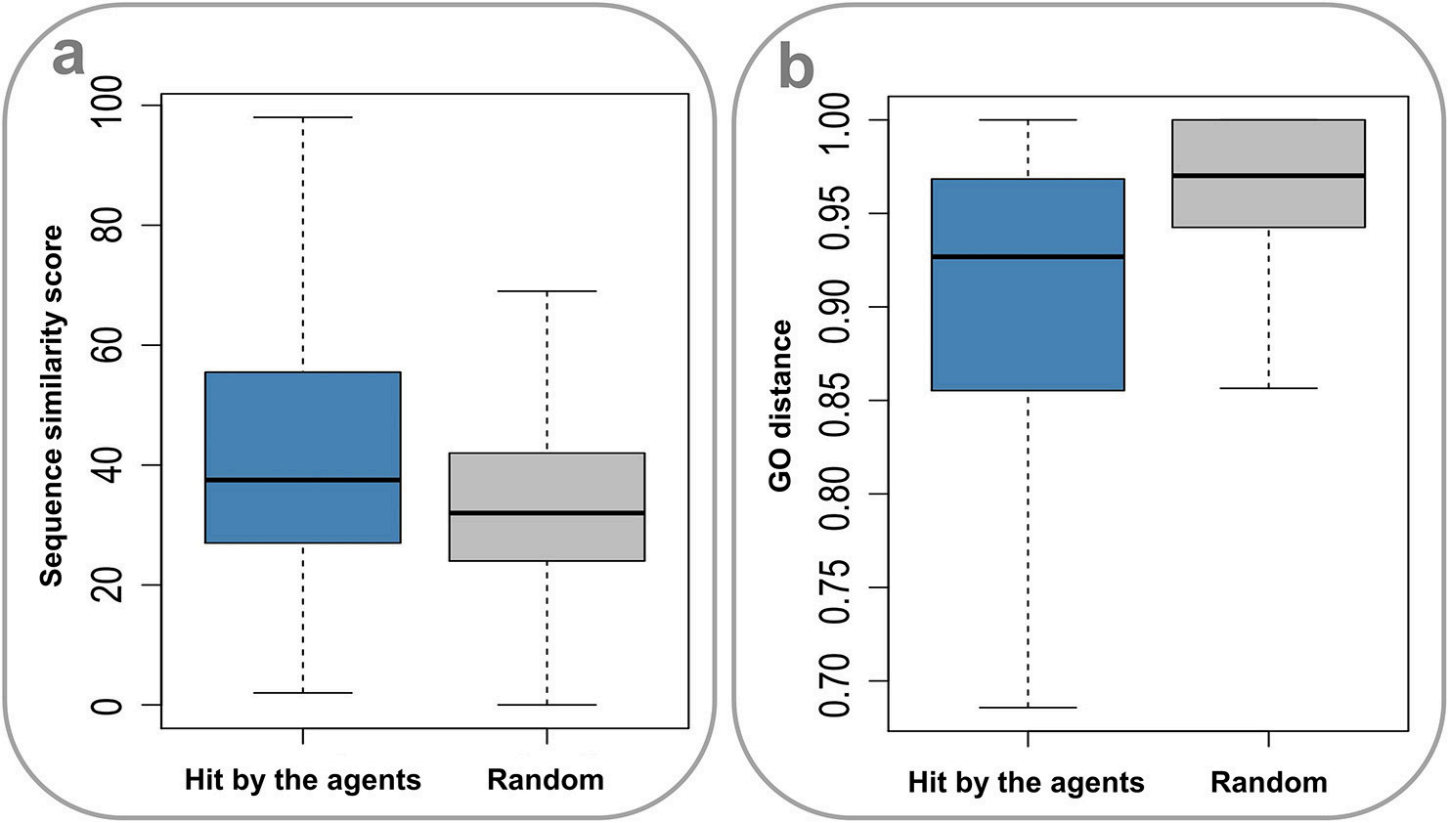
Sequence similarity and GO distances of gene pairs targeted by the multi-target agents. **(A)** The sequences for target pairs hit by the agents are more similar than those randomly selected from the target set (*P* = 2.20 × 10–16, Wilcoxon rank-sum test). **(B)** The GO-based Czekanowski–Dice distances of the gene pairs targeted by the agents are evidently smaller than those of randomly selected target pairs (*P* = 2.20 × 10–16, Wilcoxon rank-sum test).

Drug action is usually considered a specific process. It is thus of apparent interest to investigate the molecular mechanisms underlying the promiscuity of the multi-target agents. Considering the fact that human genes generate a large number of paralogs during evolution, a primary explanation is that the multiple targets covered by the agents have similar sequences and functions. Indeed, the sequences for target pairs hit by the agents are more similar than those randomly selected from the target set (*P* = 2.20 × 10^−16^, Wilcoxon rank-sum test) (Figure 4A), where the needle program of EMBOSS package (Rice et al., 2000) was used to do pairwise alignments. Furthermore, it was found that the target pairs covered by the agents are significantly enriched with paralogs (4.72% (2,602 of 55,110), derived from Ensemble database), compared with the randomly combined target pairs (0.10% (4,029 of 3,955,078), *P* ~ 0, hypergeometric test). Besides, the GO-based Czekanowski–Dice distances (Ovaska et al., 2008) of the gene pairs targeted by the agents are evidently smaller than those of randomly selected target pairs (*P* = 2.20 × 10^−16^, Wilcoxon rank-sum test) (Figure 4B). These observations not only support the evolutionary explanation to the molecular basis of multi-target drug action, but also provide useful clues to addressing the concerns about the side effects of promiscuous agents.

Despite the achievements of multi-target strategy for drug discovery, questions concerning security remain, as the tendency to act on multiple genes may increase the probability of inducing adverse effects. The present analyses indicate that these agents prefer to target genes with similar sequences and functions, namely paralogs, which means that the agent-targeting process is not so random that it will constrain the agent activities into a relatively narrow range. This is definitely beneficial to alleviate the side effects of multi-target agents and thus helpful to enhance their druggability.

**Figure 5:**
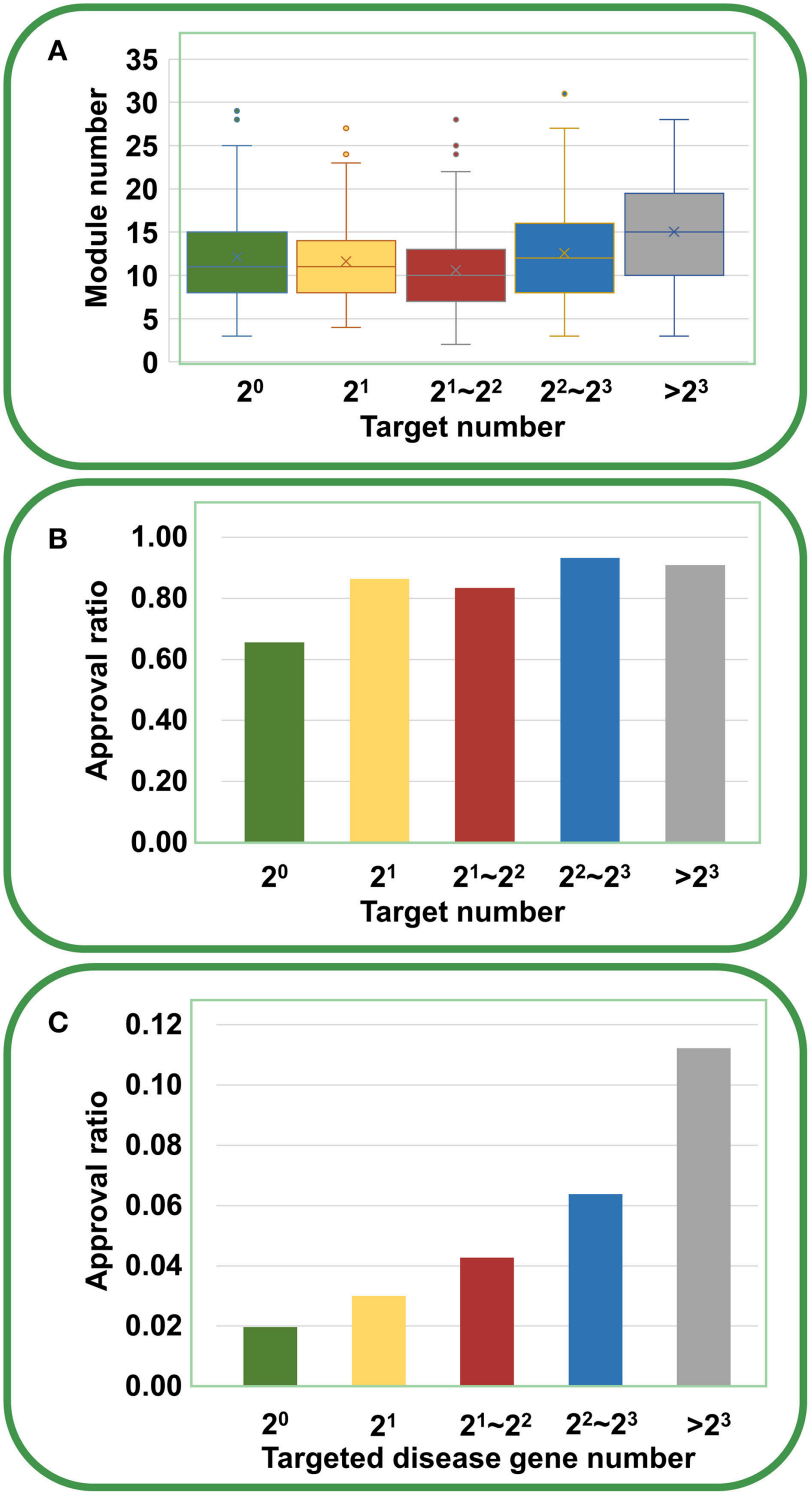
Relationships between druggability and target number of agents derived from cMap. **(A)** With the increasing number of targets, the agents cover more gene modules (ANOVA: *P* = 1.94 × 10^−9^). **(B)** With the increasing number of targets, the drug approval ratio increases slightly. **(C)** If only disease-associated genes are considered, the drug approval ratio rises evidently with the increase of targeted gene number.

Furthermore, we analyzed the chemical genetic data recorded in connectivity map (cMap) (Lamb et al., 2006). The cMap comprises 7,056 gene expression profiles for five human cell lines treated with 1,309 agents. Using the biclustering approach FABIA (factor analysis for bicluster acquisition), we have generated 49 gene modules for cMap data, establishing links between gene modules and chemical agents (Xiong et al., 2014). Therefore, each agent has a gene module profile, and the promiscuity of the agent increases with the increasing number of modules the agent covers. As shown in Figure 5A, with the increase of targets, the agents indeed cover more gene modules, supporting the opinion that multi-targeted agents have a higher risk of yielding unwanted effects. However, the druggability analysis indicated that with the increasing number of targets, the drug approval ratio does not decrease but rather increases slightly (Figure 5B). Moreover, if only disease-associated genes are considered, the drug approval ratio increases evidently with the increase of targeted gene number (Figure 5C). This observation strongly suggests that despite the enhanced risk in side effects, multi-targeted agents are still very promising in drug development.

### Dependence of Agent Activity/Druggability on Target Quality

**Figure 6:**
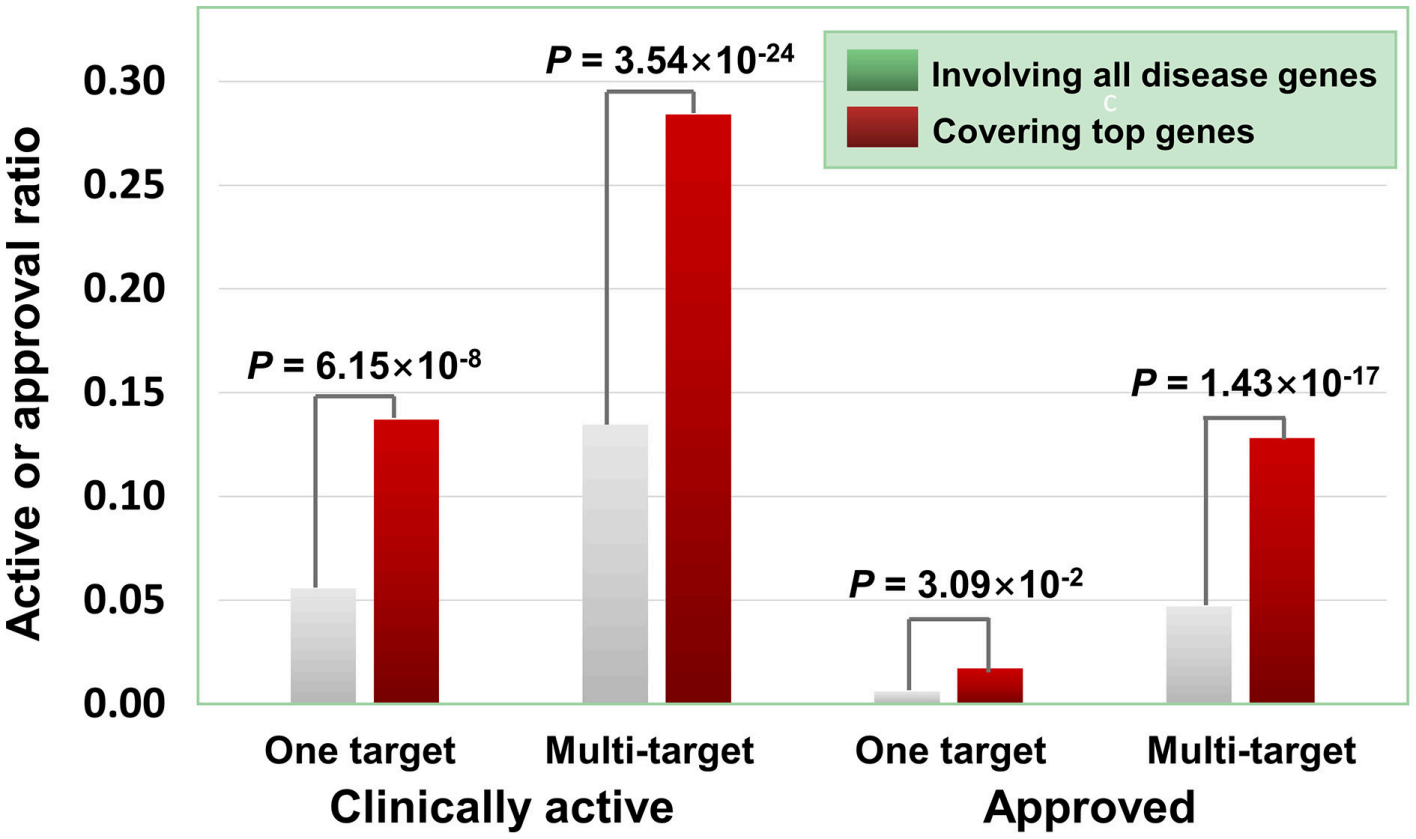
Effects of top genes on the clinically active/approval ratio of agents. The top genes were derived from AlzGene, SZGene, PDGene, and MSGene. From DGIdb, TTD and DrugBank, we retrieved 3,692 agents targeting the genes contained in the four databases, of which 726 targeted at least one top gene. The results show that for the agents covering top genes, their genetics-implicated activities are more likely to be supported by clinical trials and to be clinically approved (*P*-values were calculated using the hypergeometric test).

Besides the quantity of agent targets, their quality also influences the medicinal potential of agents in principle. Our prior study has revealed that the agents targeting “top genes” have higher therapeutic potential (Quan et al., 2018), where “top genes” were defined as those tightly associated with certain diseases. Four disease-gene databases, i.e., AlzGene (Bertram et al., 2007), SzGene (Allen et al., 2008), PDGene (Lill et al., 2012), and MSGene (Lill et al., 1994), provide “top genes” annotations for Alzheimer's disease, schizophrenia, Parkinson's disease, multiple sclerosis, respectively. From DGIdb, TTD and DrugBank, we retrieved 3,692 agents targeting the genes including “top genes” contained in these four databases (Table S2). As illustrated in Figure 6, multi-target agents exhibit higher medicinal potential than single-target counterparts, consistent with the above observations. Next, for the agents covering “top genes,” their genetics-derived activities are more likely to be supported by clinical evidence and be clinically approved (Figure 6 and Table S2), indicating the importance of target quality in genetics-based drug discovery.

However, only a few genetic databases contain quality information for disease genes. Considering the above finding that multi-target agents usually hit paralogs, we speculated that ohnolog genes, i.e., paralogs generated by whole genome duplication, may be used as “top genes” instead. Ohnolog genes have been recognized to significantly enrich disease genes, compared with other paralog genes, because of their strong dosage balance (Makino and Mclysaght, 2010; McLysaght et al., 2014; Xie et al., 2016; Sekine and Makino, 2017).

**Figure 7:**
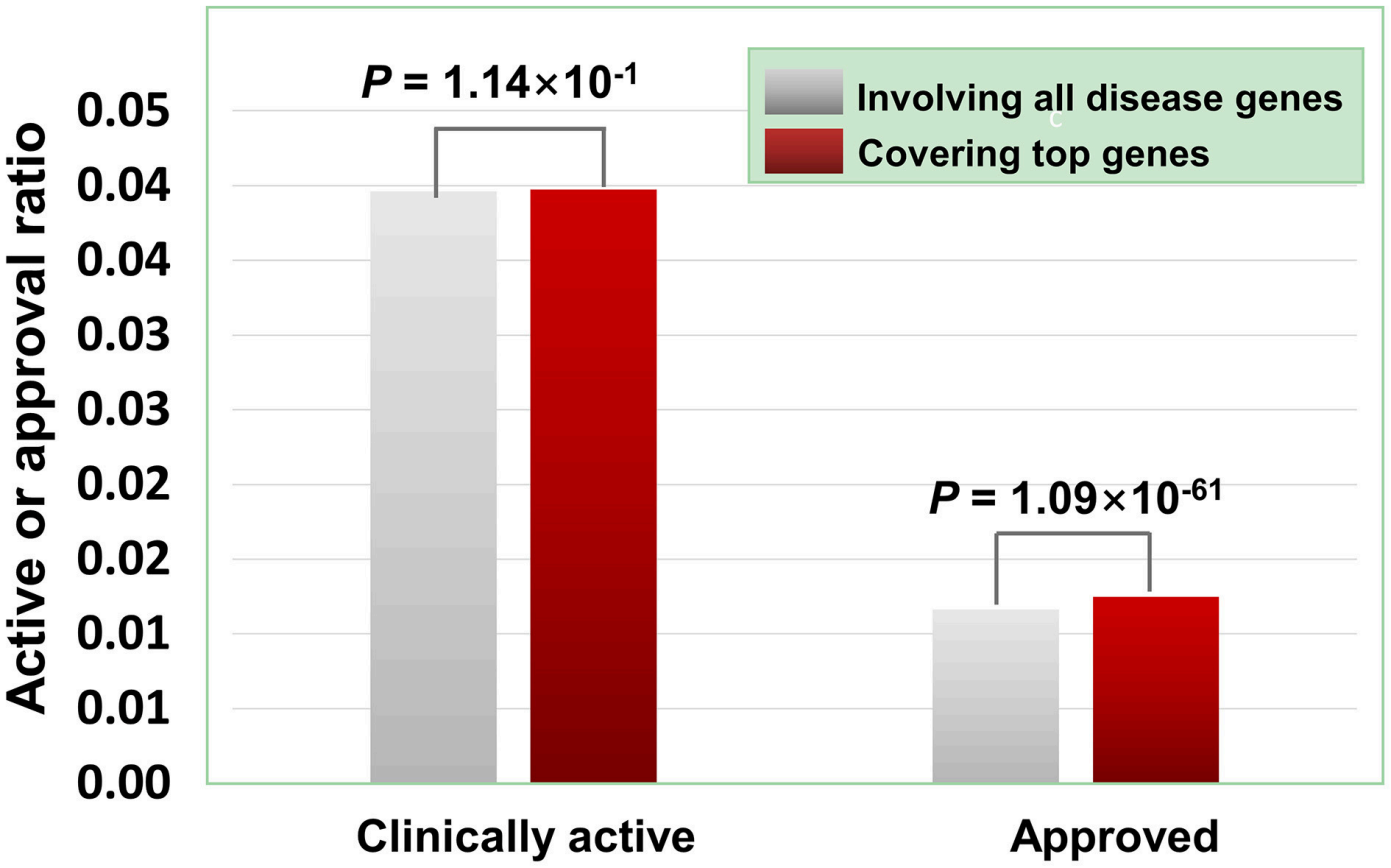
Effects of disease-associated ohnolog genes on the clinically active/approval ratio of agents. A total of 7,294 ohnolog genes were obtained from Makino and Mclysaght's work31, in which 5,265 genes were disease-associated. Searching DGIdb, TTD and DrugBank revealed that 4,058 agents targeted 1,164 of the 5,265 ohnolog genes. The results show that for the agents covering disease-associated ohnolog genes, their genetics-derived activities are more likely to be supported by clinical evidence and be clinically approved (*P*-values were calculated using the hypergeometric test).

As illustrated in Figure 7, the agents covering disease-associated ohnolog genes indeed exhibit higher approved potential (*P* < 1.09 × 10^−61^, hypergeometric test), suggesting that disease-associated ohnolog genes can be regarded as “top genes” to some extent. This finding is very useful in establishing the machine-learning models for drug activity prediction (see below for details).

### Target Quality Evaluation and Druggability Score of Disease Genes

**Figure 8:**
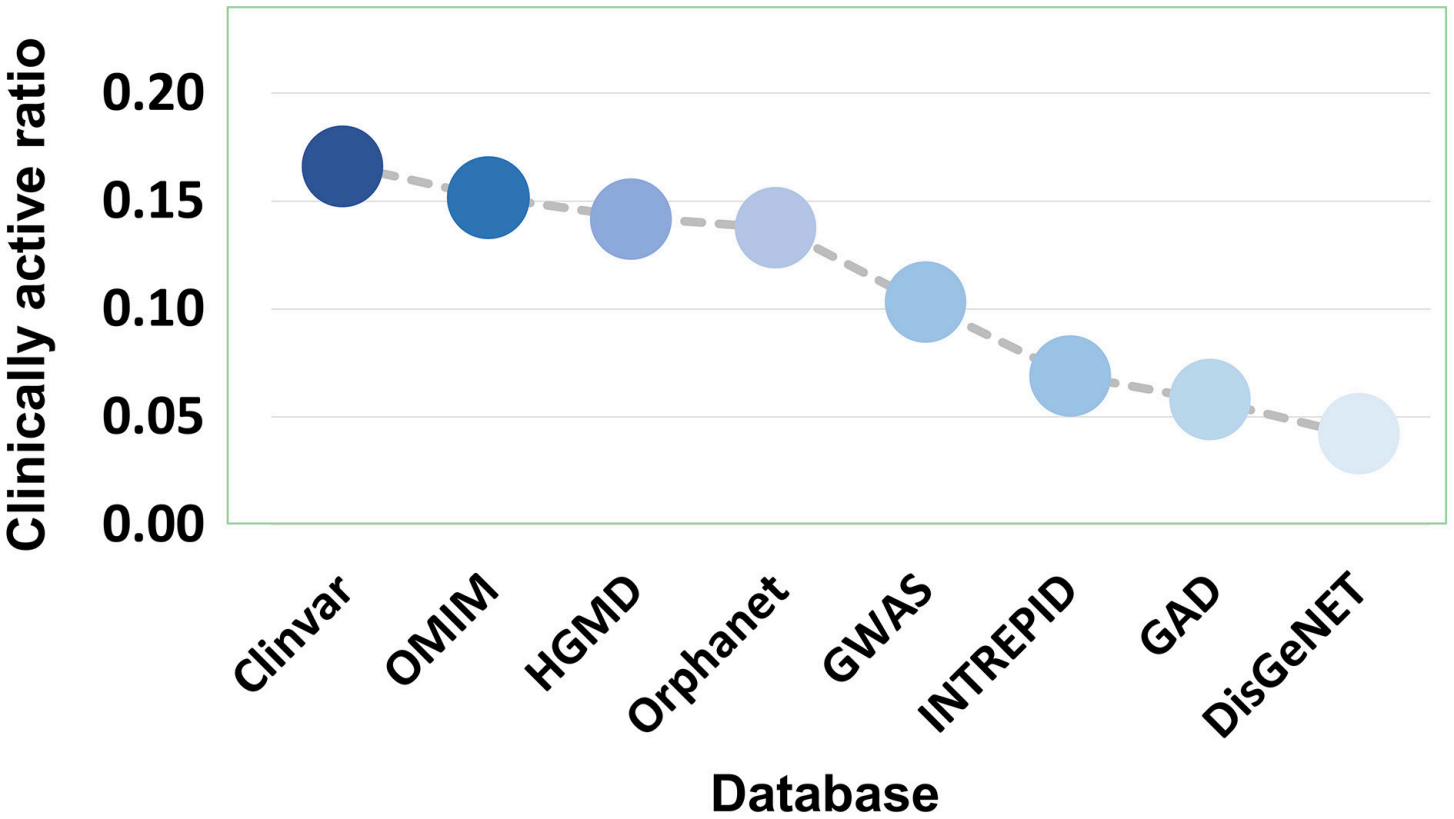
Clinically active ratio of genetics-implicated agent indications derived from different disease gene databases.

Eight disease gene databases (including Clinvar, OMIM, HGMD, Orphanet, GWASdb, INTREPID, GAD, and DisGeNET) are used in the present study. The target quality of each database must be different, which stimulated our interest to do an evaluation by comparing the clinically supported ratio of genetics-implicated agent activities derived from eight databases. The results showed that target genes of Clinvar have the highest quality, in which 16.52% of genetics-based activity predictions are supported by clinical test. The target quality (measured by clinically active ratio) of other databases declines in the order: OMIM (15.01%), HGMD (14.09%), Orphanet (13.62%), GWASdb (10.53%), INTREPID (7.08%), GAD (5.75%), and DisGeNET (4.14%) (Figure 8 and Table S3). This observation inspired us to propose a parameter for quantitatively measuring the druggability of disease genes. First, the genes derived from different databases were given different quality scores, with the highest-quality database (i.e., Clinvar) being assigned with the highest score (eight points), while the lowest (i.e., DisGeNET) with the lowest score (one point). Then, the scores were summed up for each disease gene to define its druggability (**see Methods**). The higher the score is, the more druggable the disease gene. Apparently, a gene may have different scores for different diseases.

**Figure 9:**
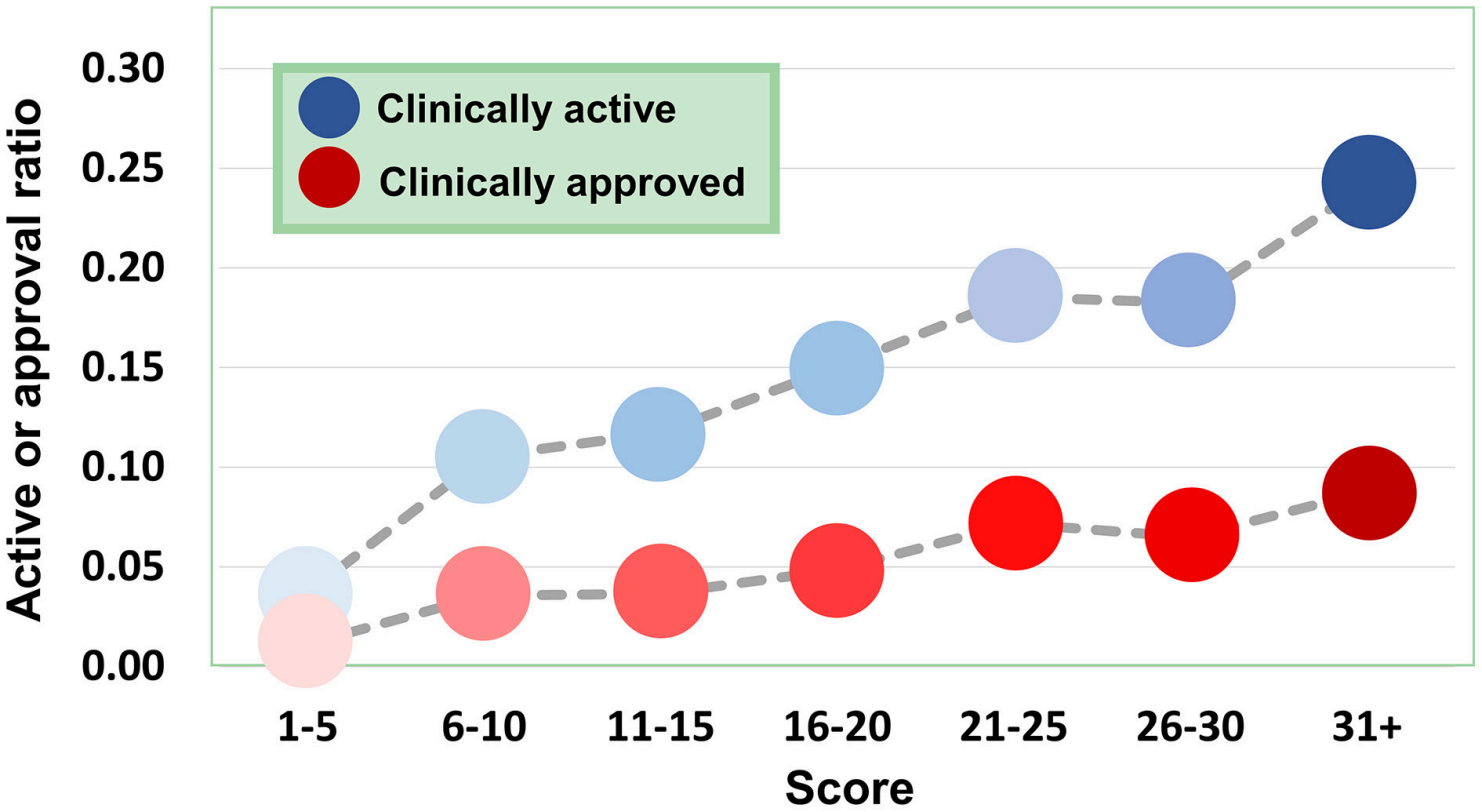
Dependence of agent activity/druggability on target quality. With the increase of druggability scores of target genes, the therapeutic potential of corresponding agents also increases.

**Figure 10:**
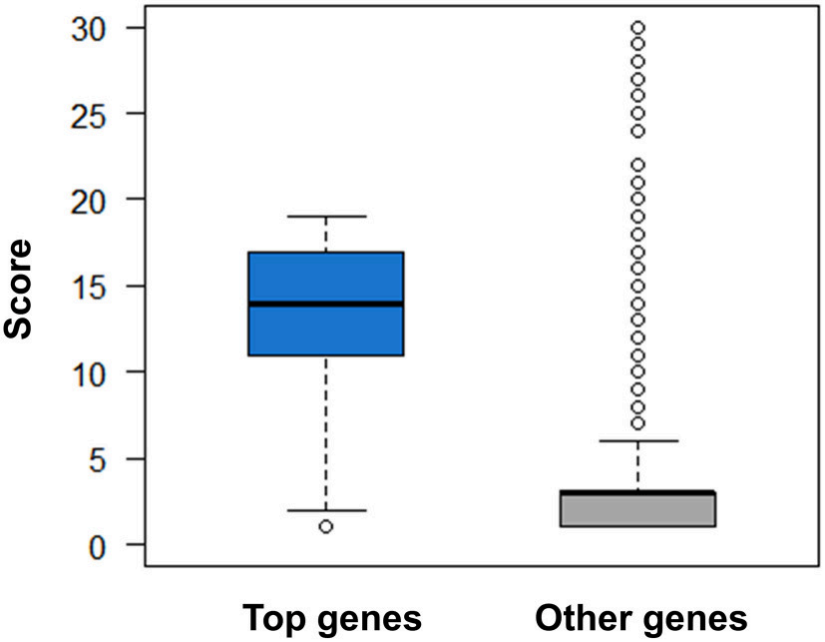
Comparison of druggability scores for top genes derived from AlzGene, SzGene, PDGene, MSGene, and ordinary genes with the same pathogenic annotations. The top genes exhibit evidently higher scores than other genes (*P* = 2.51 × 10–52, Wilcoxon rank-sum test).

**Figure 11:**
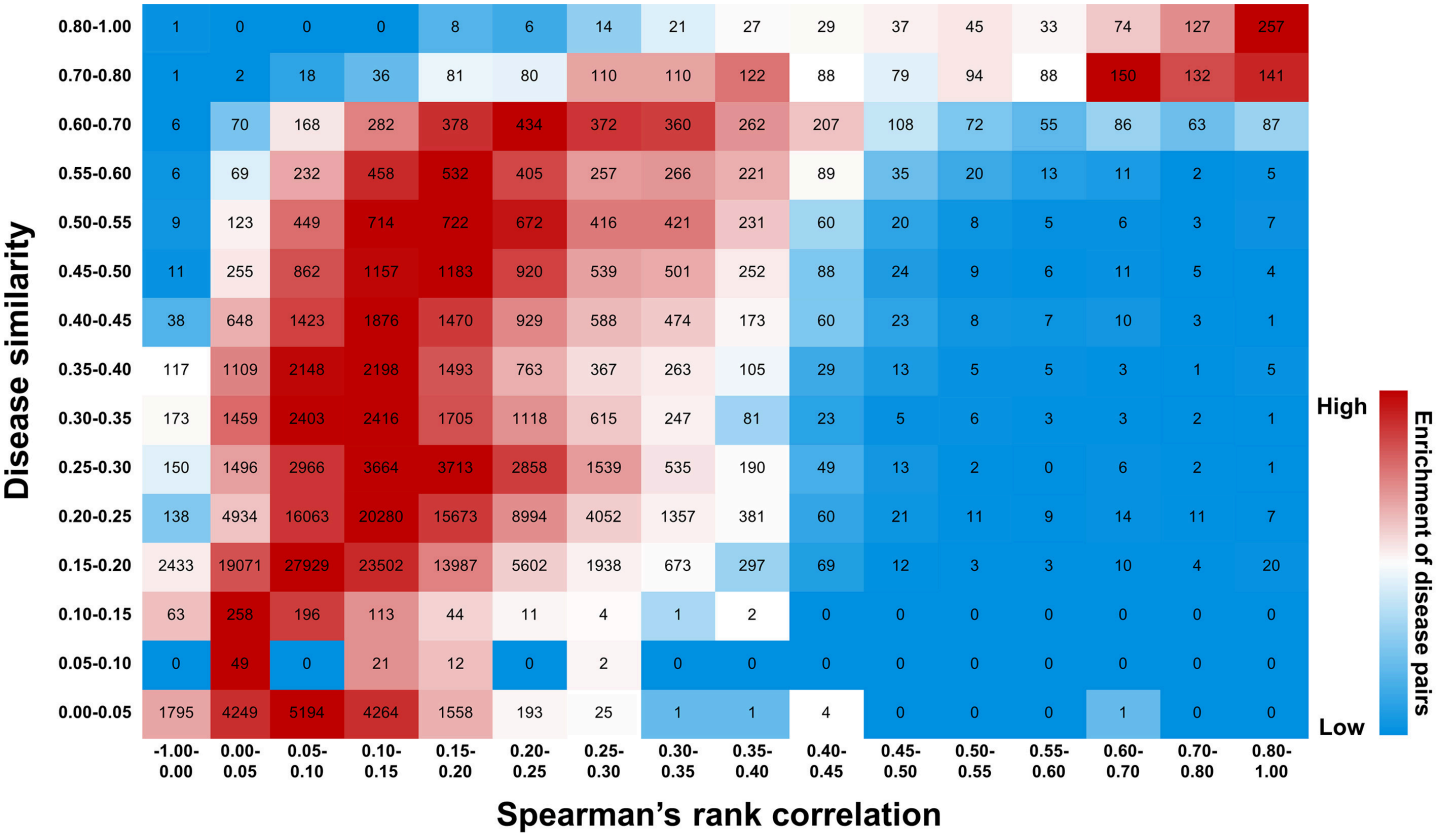
Efficiency of druggability scoring system in characterizing gene-disease links. Diseases exhibiting similar gene profiles, calculated by Spearman's rank correlation, display similar symptoms measured by UMLS::similarity. The number of disease pairs is shown in the box. The color exhibits enrichment of the number in each row, with red representing the strong enrichment and blue representing the weak.

This scoring system is validated by the following observations. First, for the disease genes with higher druggability scores, the genetics-implicated activities of agents are more possible to be clinically supported and approved (Figure 9 and Table S4). Considering the correlation between gene druggability and pathogenicity (Plenge et al., 2013; Quan and Zhang, 2016), it is inferred that druggability score is also appropriate for characterizing gene-disease links. Indeed, the “top genes” derived from AlzGene, SzGene, PDGene, and MSGene, which are tightly connected with diseases, exhibit much higher druggability scores than other genes with the same pathogenic annotations (*P* = 2.51 × 10^−52^, Wilcoxon rank-sum test) (Figure 10). Therefore, each disease can be characterized by the corresponding scored genes, constituting a gene profile pertinent to the disease. Different diseases can be compared through calculating Spearman's rank correlation between their gene profiles. It is interesting to notice that the diseases exhibiting similar gene profiles display similar symptoms measured by UMLS::similarity (Figure 11), validating the scoring system in characterizing gene-disease links. Together, it is concluded that druggability score can be used to measure target quality and genetic links between genes and diseases, which is of great value in drug activity prediction by machine-learning models.

### Agent Activity Prediction With Multi-Label Classification Model

The above analysis implied that it is possible to establish drug-activity prediction models based on the genetic information of drug targets. Since a drug is usually associated with multiple activities for diseases and a disease could be treated by multiple drugs, drug-activity prediction problem can be considered as a multi-label classification task. In this paper, we adopted a method of multi-label k-nearest neighbor (MLKNN) which can construct high-accuracy multi-label prediction models for drug-activity prediction (Zhang and Zhou, 2007; Wen et al., 2015).

First, we investigate a variety of features to represent the characters of druggability. Considering that various features may bring diverse information as well as noise, we adopt ensemble learning method to select suitable features to build the models (Lee and Soo, 2013; Yang et al., 2014; Zhang et al., 2015). Considering that agents targeting multiple disease genes, in particular “top disease genes” and genes with high druggability scores, tend to show high therapeutic potential (Figures 3, 6, 7, 9), we rationally selected four parameters to build the models. The first parameter characterizes the overall score of genes responsible for certain diseases within drug targets, and the second parameter is the normalized average value of the overall score. The third and fourth parameters describe the absolute number and relative ratio of ohnologous disease genes (serving as “top genes”) within drug targets, respectively (**see Methods**).

Representation of drug labels is a crucial step in multi-label learning. An agent-disease pair was regarded as a positive, if the drug hits one or more disease genes and is indicated for treating this disease. An agent-disease pair was regarded as a negative, if the drug targets one or more disease genes but is not annotated for controlling this disease. As a result, a total of 74,902 positives covering 5,759 agents and 667 diseases, and 3,778,517 negatives were selected.

Given a dataset of n drugs denoted as ${\left\{\left(x_{i},y_{i}\right)\right\}}_{i=1}^{n}$, *x*_*i*_ and *y*_*i*_ are the *p*-dimensional feature vector and *q*-dimensional disease vector for the *i*th drug, respectively. Our goal is to build the functional relationship *Y* = *F*(*X*):2^*p*^ → 2^*q*^ between exploratory variables (feature vector) and target values (agent-activity vector) for multi-label learning.

First, four MLKNN models were constructed based on four features. Then, each model was evaluated by the internal 5-fold cross validation on the training data. As a result, five MLKNN models were built based on five internal folds and selected features. The final prediction result is the average and standard deviation scores of outputs by five MLKNN models. At last, we used the ensemble learning method to combine four features and generate high-accuracy prediction models **(see Methods**).

**Figure 12:**
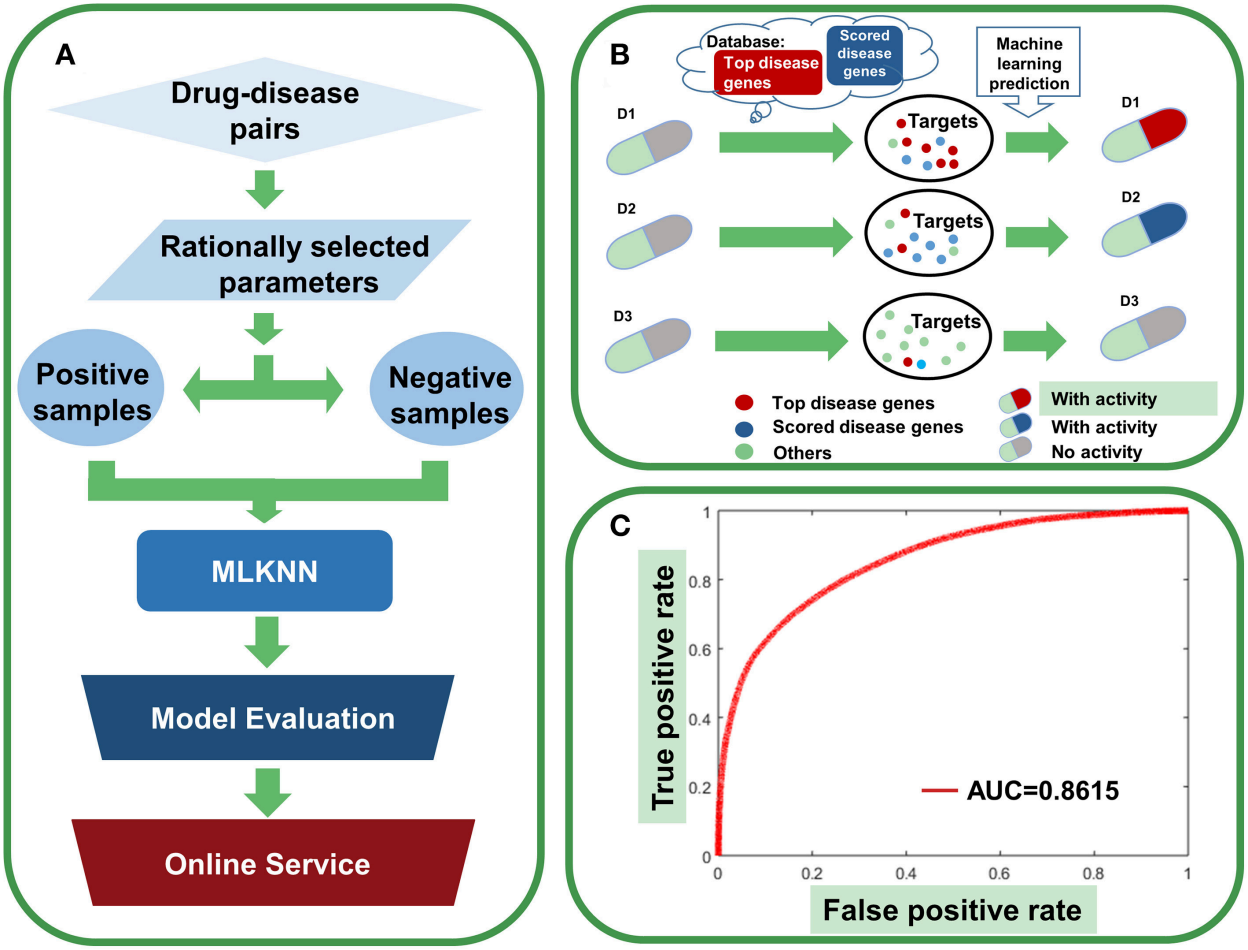
Agent activity prediction with machine-learning models. **(A)** Workflow for the machine-learning model establishment. **(B)** Sketch view for the rationale of agent activity prediction. **(C)** The overall performance of the ensemble classifier.

The performance of assembled classifier for agent-activity prediction is shown in Figure 12. For a 5-fold stratified cross-validation with a 1,000 repeat, MLKNN displays the best performance (Table S5). By inputting the 5,759 original agents and associated targets into the models (where the threshold of predictive value was set to 0.5), 11,649 activities were predicted. 67.01% of the predicted activities are supported by clinical trials, and 14.52% have been approved, which are much higher than the overall ratio of genetics-implicated clinical activity and approved indication (3.96 and 1.16%, respectively).

**Figure 13:**
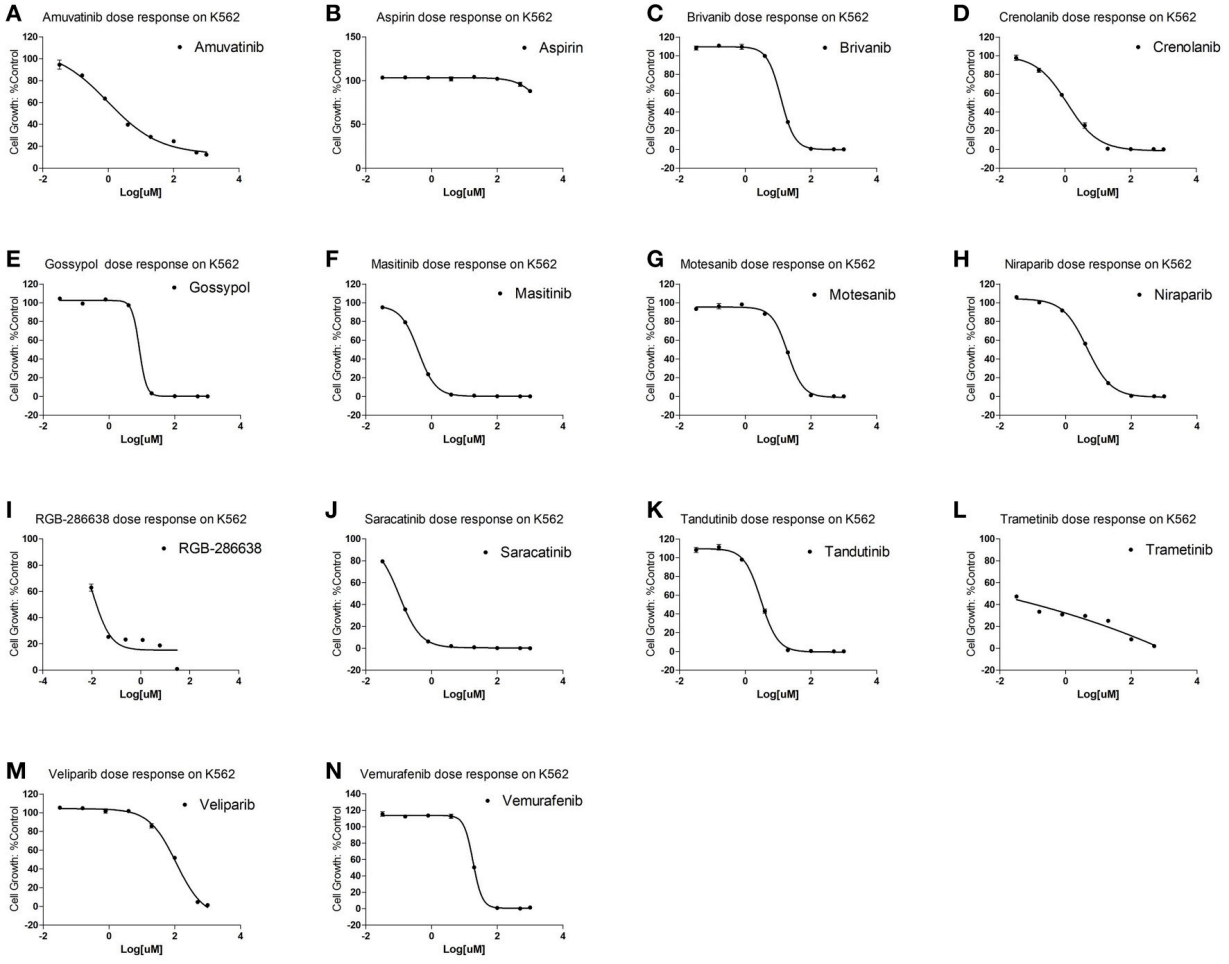
Cytotoxicity of 14 predicted anti-leukemia agents. K562 cells were treated with **(A)** Amuvatinib, **(B)** Aspirin, **(C)** Brivanib, **(D)** Crenolanib, **(E)** Gossypol acetic acid, **(F)** Masitinib, **(G)** Motesanib, **(H)** Niraparib, **(I)** RGB-286638, **(J)** Saracatinib, **(K)** Tandutinib, **(L)** Trametinib, **(M)** Veliparib, **(N)** Vemurafenib. The results show that 10 agents (Amuvatinib, Brivanib, Crenolanib, Masitinib, Motesanib, Niraparib, Saracatinib, Tandutinib, Veliparib, Vemurafenib) (71.43%) can efficiently inhibit the growth of K562.

To examine for which kind of diseases the predictions are most relevant, we compared the clinically active/approval ratio of the predicted results for various diseases. It was found that, leukemia and lymphoma have the most predictions (Table S6). To demonstrate the usefulness of the present method, we tested the predicted anti-leukemia agents by cytotoxicity experiment. Using our models, 809 agents were predicted to have anti-leukemia potential, of which 550 (67.99%) have been validated by prior clinical tests. Thus, it is intriguing to examine the anti-leukemia potential of the rest 259 agents. 14 of 259 agents are commercially available, which were evaluated by K562 (chronic myeloid leukemia-derived cancer cell line) cytotoxicity assays. The results show that 10 agents (71.43%) can inhibit the growth of K562 efficiently (Figure 13) (Table S7), with IC50 values ranging from 0.106 (saracatinib) to 111.2 μM (veliparib) (Table S7).

To facilitate the use of the machine-learning prediction models, we developed a web server SCG-Drug (Systems Chemical Genetics-Drug, http://zhanglab.hzau.edu.cn/scgdrug) that allows a quick and intuitive access to the background information and predicted results. Currently, SCG-Drug contains 5,759 agents, 703 diseases and 19,233 genes derived from various databases. By inputting the target information of any agents into SCG-Drug, one can use the established machine-learning models to predict the potential activities of the agents. The SCG-Drug web interfaces allow users to explore medicinal information related to a given drug, disease or gene through four interfaces in “Analysis” page: “Drug”, “Batch prediction,” “Disease,” and “Gene.” The “Drug” interface allows users to submit a single drug to retrieve target genes and potential activities of the query drug. For example, when a user submits a single drug that was shown in the dropdowns, the drug will be searched in the database directly. If it is unable to find any matches for the search term, the user will be asked to input the corresponding target genes of the drug. Then, the system will call the prediction module. Alternatively, the system allows the user to upload a file on the “Batch prediction” interface, in which an agent and corresponding targets are in a single row and the terms in each row are separated by tabs, along with an email address to which the predicted activities of the agents will be sent. Offline prediction automatically starts, and the predicted results will be sent to the user via e-mail. The “Disease” interface allows users to obtain relevant disease genes with druggability score, and database source by querying standardized disease descriptions of MeSH. The “Gene” interface allows users to explore gene-related diseases (with druggability score) and drugs only by submitting a gene name or an Entrez ID, which have been documented in the server. In addition, users can obtain the information for documented drugs (with normalized indications) and targets/genes (with normalized disease descriptions) from “Download” page. The data and the machine-learning models will be updated regularly.

## Discussion

Selecting agents with desired activities and high druggability from an infinite chemical space is a fundamental task for drug development. Previous studies have revealed that genetic disease genes can provide valuable clues for drug activity prediction and druggability assessment (Brinkman et al., 2006; Sanseau et al., 2012; Wang Z. Y. et al., 2012; Plenge et al., 2013; Wang and Zhang, 2013; Okada et al., 2014; Nelson et al., 2015). However, these studies are limited to single-drug-single-target paradigm. Because most complex diseases are caused by multiple pathogenic factors, it is reasonable to speculate that targeting multiple disorder factors will better navigate the drug space. In this study, by a comprehensive analysis, we clearly indicate that aiming at multiple disease genes is helpful to prioritize drug candidates with promising activities and high druggability. Additionally, the strengthened genetic links between target genes and diseases are helpful to improve the medicinal potential of drug candidates. The drug-gene interaction information is expected to be rapidly accumulated through emerging techniques in chemical biology. However, the identification of reliable genetic links between genes and diseases depends on progress in medical genetics.

A number of systems genetics methods have been developed for enriching and screening the driver genes underlying complex traits in the post-GWAS era. For example, Zhu et al. identified 126 genes related to human complex traits through the integration of summary-level GWAS results and eQTL data (Zhu et al., 2016). Based on the exome sequencing, array copy number and RNA sequencing (RNA-seq) data from 3,281 samples across 12 cancer types, Leiserson et al. performed a pan-cancer analysis of mutated networks utilizing a HotNet2 (HotNet diffusion-oriented sub-networks) algorithm, by which they identified 16 significantly mutated subnetworks containing 147 genes. Many of these genes have been validated to play a critical role in cancer pathogenesis (Leiserson et al., 2015). Gamazon et al. proposed a gene-based association method called PrediXcan that directly tests the molecular mechanisms through which genetic variation affects phenotype (Gamazon et al., 2015). Greene et al. introduced a Network-guided GWAS Analysis method called NetWAS, which integrated tissue-specific networks and nominally significant *P*-values in GWAS to identify biologically important disease-gene associations (Greene et al., 2015). Although these methods are helpful to identify reliable genes associated with a complex disease trait, the complex application procedures hinder their convenient use. In this study, we endorsed the possibility of using ohnolog genes as a source of “top disease genes.” The high accessibility of ohnologs will facilitate the identification of disease driver genes and the genetics-based drug discovery.

The above discoveries inspired us to establish systems chemical genetic models for predicting drug activities. Because drug repurposing is a hot spot in the pharmaceutical industry, a number of theoretical methods, including cheminformatics-based, bioinformatics-based and systems biology-based methods, have been proposed to predict drug activities (Jin and Wong, 2014). However, most of these methods were derived from parameters trained using large datasets, suggesting that these methods may be sensitive to datasets and poor in generalization capabilities. The identification of the genetic determinants of drug activities facilitates the rational selection of parameters to establish machine-learning models for drug activity prediction. Because this model was built on the fundamental principle of drug activity determination, it is expected to be robust when generalized to different datasets and explainable to certain extent. Moreover, to maximize the convenience for researchers, a user-friendly online service (SCG-Drug) was provided for drug-activity prediction and data retrieval as well. These systems chemical genetics methods are of high value in prioritizing drug candidates, also highlighting the importance of modern genetics in facilitating the paradigm shift of pharmaceutical industry.

## Materials and Methods

### Data Sources and Pre-processing

#### Agent Information

We collected agents and agent-target associations from three databases: DrugBank, TTD, and DGIdb (Law et al., 2014; Qin et al., 2014; Wagner et al., 2015). By integrating the 6,841 agents covering 3,692 targets from DrugBank, the 5,208 agents covering 569 targets from TTD, and the 10,941 agents covering 3,090 targets from DGIdb, we obtained 35,860 agent-target associations, comprising 16,021 agents and 4,613 target genes. The indication information for the agents were collected from DrugBank, TTD, and ClinicalTrials (Zarin et al., 2011; Law et al., 2014; Qin et al., 2014). Totally, we obtained 80, 90 agents with corresponding target genes and pharmacological activity records. Using the disease classes provided by Pharmaprojects (similarity threshold: 0.75, for more details see the ***Disease standardization***section), we finally acquired 5,759 agents covering 667 types of diseases and 2,813 target genes.

#### Disease-Associated Genes

Eight databases were used to collect disease-related genes, including the Genetic Association Database (GAD, https://geneticassociationdb.nih.gov/) (Becker et al., 2004), Online Mendelian Inheritance in Man (OMIM, http://omim.org/) (Hamosh et al., 2005), Clinvar (http://www.ncbi.nlm.nih.gov/clinvar/) (Landrum et al., 2014), Orphanet (http://www.orpha.net/consor/cgi-bin/index.php), DisGeNET (http://www.disgenet.org/web/DisGeNET/menu/rdf) (Piñero et al., 2015), INtegrated TaRget gEne PredItion (INTREPID) (Chen and Tian, 2016), GWASdb (http://jjwanglab.org/gwasdb) (Nelson et al., 2015) and The Human Gene Mutation Database (HGMD, http://www.hgmd.cf.ac.uk/ac/index.php) (Wang X. et al., 2012). A total of 19,233 disease-associated genes were collected for use in the present analysis. Genes that could not be mapped to an Entrez ID were excluded. The available URLs, version information, access dates, and number of records from the above eight databases are provided in Table S8.

#### Disease Standardization

We used the Unified Medical Language System (UMLS), which provides a comprehensive set of medical concepts, to standardize disease annotations of genes, and agents. UMLS is a medical terminology system that has been developed by the National Library of Medicine for more than 20 years and contains a large number of standardized medical concepts. The natural language processing program MetaMap was used to convert disease annotations to the corresponding disease concepts (Aronson, 2001). We selected Medical Subject Headings (MeSH) as the vocabulary, and limited the semantic type to “Pathologic Function,” “Injury or Poisoning,” and “Anatomical Abnormality” to obtain the disease-related concepts (Liu et al., 2014). We processed all gene-related phenotypes and agents' indications using the UMLS concept. As MeSH defines disease concepts using a hierarchical system, it classifies each disease to a narrow disease type; for example, “Alzheimer disease 15” is a subtype of “Alzheimer disease.” The latter is simply a broader term for the former. In our work, all subtype disease concepts were converted to the appropriate broader term using a Perl module UMLS::Interface. Disease annotations that could not be mapped to any disease concept were excluded from subsequent analyses. Using the disease classes provided by Pharmaprojects (similarity threshold: 0.75) (Mcinnes et al., 2009), we obtained 914,190 gene-disease pairs (involving 703 types of diseases) and 74,902 agent-disease pairs (involving 667 types of diseases).

#### Sequence Similarity Analysis

The needle program of EMBOSS package (Version: 6.6.0.0) (Rice et al., 2000) was employed to perform sequence similarity analysis of agent-targeted proteins, because of its accurate production of Needleman-Wunsch global pairwise alignments.

#### Gene Ontology (GO) Terms Similarity Measurement

We used the GO-based Czekanowski–Dice distance to evaluate the GO terms similarity of the target pairs. The Czekanowski–Dice functional distance was calculated using a previously described method (Ovaska et al., 2008). The GO term information of the gene pairs was obtained from the Ensembl database (version 72).

#### “Top Genes” and Ohnolog Genes

The AlzGene database contains 650 genes for Alzheimer's disease (Bertram et al., 2007); the SzGene database contains 937 genes for schizophrenia (Allen et al., 2008); the PDGene database contains 571 genes for Parkinson's disease (Lill et al., 2012); and the MSGene database contains 675 genes for multiple sclerosis (Lill et al., 1994). From these databases, 44, 43, 31, and 43 genes strongly associated with Alzheimer's disease, schizophrenia, Parkinson's disease and multiple sclerosis, respectively, were identified. These genes were termed “top genes,” meaning that relatively reliable associations have been established between these genes and certain diseases. In addition, the ohnologs served as an alternative source of “top disease genes,” because ohnologs are significantly enriched with disease genes due to their strong dosage balance (Makino and Mclysaght, 2010; McLysaght et al., 2014). From Makino et al.'s work (Makino and Mclysaght, 2010), we extracted 9,057 ohnolog pairs covering 7,295 genes from the human genome.

#### Druggability Score of Disease Genes

Based on clinically active ratio of genes from eight disease databases (Clinvar, OMIM, HGMD, Orphanet, GWASdb, INTREPID, GAD, and DisGeNET), we proposed a parameter named druggability score for quantitatively measuring the druggability of disease genes. First, the genes derived from different databases were given different scores, with the highest-clinically active ratio database (i.e., Clinvar) being assigned with the highest score (eight points), the disease genes obtained from the second-ranked database of the clinically active ratio (i.e., OMIM) was given seven points, and so on, from HGMD was given six points, from Orphanet was given five points, from GWASdb was given four points, from INTREPID was given three points, from GAD was given two points, while the lowest clinically active ratio (i.e., DisGeNET) with the lowest score (one point) (Table S3). Then, if a disease gene is recorded in multiple databases, the scores of the corresponding multiple databases were summed up for this disease gene to define its druggability:

(1) $$\begin{array}{l} Druggability\;score=\sum_{j=1}^{k}score_{ij} \end{array}$$

where *score*_*ij*_ denotes the assigned score of a pathogenic gene *i* in the jth database (Table S3); *i* = 1, 2, …, *m*; *j* = 1, 2, …, *k*, where *m* is the number of disease genes, *k* is the number of databases (*k* = *8* in this study).

### Statistical Analysis

#### Disease Similarity Measurement

First, the disease terms of genes and indication annotations of agents were converted to the standardized medical concepts of UMLS by a natural language processing tool MetaMap. Then, through using the disease classes provided by pharmaprojects (Similarity threshold: 0.75), the disease similarity was measured using UMLS::similarity. Lin, which is calculated using the information content and path of concepts, shows good performance for disease similarity measurement (Nelson et al., 2015). In this study, we used the Lin to evaluate the disease term similarity of all disease concepts. The Lin is calculated using the following equation:

(2) $$\begin{array}{l} Lin=\;\frac{IC\left(lcs\right)}{IC\left(concept1\right)+IC\left(concept2\right)} \end{array}$$

where *IC* is the negative log of the probability of the concept, the probability is pre-calculated by the Perl module by summing the probability of the concept occurring in some text plus the probability of its descendants occurring in some text, and *lcs* is the least common subsuming concept of concept1 and concept2.

#### Tanimoto Coefficient Calculation

To assess the correlations between disease concepts and their corresponding causal genes or drugs, we characterized the distance between disease gene sets or drug sets using the Tanimoto coefficient. The Tanimoto coefficient (*TC*) is calculated using the following equation:

(3) $$\begin{array}{l} TC=\frac{N_{AB}}{N_{A}+N_{B}-N_{AB}} \end{array}$$

where N_A_ is the number of disease A-related genes or drugs, N_B_ is the number of disease B-related genes or drugs, and N_AB_ is the number of common genes or drugs for disease A and disease B.

#### Permutation Test

To evaluate the quality of agent-target pairs, we did a 10000-permutation test on the three sets of agent-target pairs derived from DGIdb, TTD and DrugBank (Law et al., 2014; Qin et al., 2014; Wagner et al., 2015), respectively. The agents were randomly assigned with targets and the clinically active ratio of agents was calculated. This random shuffling procedure was repeated for 10,000 times.

### Machine-Learning Modeling

#### Feature Generation

We rationally selected four parameters to build the model. The first parameter characterizes the overall druggability score of the pathogenic genes within drug targets. The second parameter is the average value of the first parameter and is normalized by 36 (namely 8~). For example, if an agent targets two related disease genes derived from Clinvar and DisGeNET, respectively, the first parameter will be 9 (8 + 1), and the second parameter will be 0.125 (9/2 × 36). The third and fourth parameters are the absolute number and relative ratio of ohnologous disease genes within drug targets, respectively.

#### Positive Sample Generation

An agent-disease pair was regarded as a positive, if the drug hits one or more disease genes and is indicated for treating this disease. The positive samples were generated as 74,902 agent-disease pairs.

#### Negative Sample Generation

An agent-disease pair was regarded as a negative, if the drug targets one or more disease genes but is not annotated for controlling this disease. The negative samples were generated as 3,778,517 pairs. In the web server SCG-Drug (http://zhanglab.hzau.edu.cn/scgdrug), the model with all samples is provided.

#### MLKNN

Given the training set ${\left\{\left(x_{i},y_{i}\right)\right\}}_{i=1}^{n}$, *x*_*i*_ is the *i*th instance (drug), and *y*_*i*_ is the corresponding disease vector. *y*_*i*_(*l*) = 1. If the *i*th instance can treat the *l*th disease, otherwise *y*_*i*_(*l*) = 0, *l* = 1, 2, …, *q*. The *k* nearest neighbors (in training set) of instance *x*_*i*_ are denoted by *N*(*x*_*i*_), *i* = 1, 2, …, *n*. Thus, based on *l*th disease of these neighbors, a membership counting vector can be denoted as:

(4) $$\begin{array}{l} C_{x_{i}}\left(l\right)=\;\underset{a\in N\left(x_{i}\right)}{\sum }y_{a}\left(l\right),\;l=1,2,\ldots ,q\; \end{array}$$

where *C*_*x*_*i*__(*l*) counts the number of neighbors of *x*_*i*_ treating the *l*th disease, and 0 ≤ *C*_*x*_*i*__(*l*) ≤ *k*.

For a test drug *t*, MLKNN identifies its *k* nearest neighbors in the training set and calculate *C*_*t*_(*l*). Let $H_{1}^{l}$ be the event that a drug has *l*th disease and $H_{0}^{l}$ be the event that a drug does not treat *l*th disease. Let $E_{j}^{l}$ be the event that a drug just has *j* neighbors with *l*th disease in its *k* nearest neighbors. For the instance *t*, its label for *l*th disease *y*_*t*_(*l*) is determined by the following principle:

(5) $$\begin{array}{l} y_{t}\left(l\right)=\arg{max}_{b\in \left\{0,1\right\}}P\left(H_{b}^{l}|E_{C_{t}\left(l\right)}^{l}\right),\;l=1,2,\ldots ,q \end{array}$$

Using the Bayesian rule, above Equation (5). can be rewritten as:

(6) $$\begin{array}{l} y_{t}\left(l\right)=\arg{max}_{b\in \left\{0,1\right\}}\frac{P\left(H_{b}^{l}\right)P\left(E_{C_{t}\left(l\right)}^{l}|H_{b}^{l}\right)}{P\left(E_{C_{t}\left(l\right)}^{l}\right)}\\ \;=\arg{max}_{b\in \left\{0,1\right\}}P\left(H_{b}^{l}\right)P\left(E_{C_{t}\left(l\right)}^{l}|H_{b}^{l}\right) \end{array}$$

In the prediction model, $P\left(H_{b}^{l}\right)$ and $P\left(E_{C_{t}\left(l\right)}^{l}|H_{b}^{l}\right)$ are calculated based on the training set. The prior probabilities are calculated.

(7) $$\begin{array}{l} P\left(H_{1}^{l}\right)=\frac{\left(s+\;\sum_{i=1}^{n}y_{i}\left(l\right)\right)}{\left(s\times 2+n\right)}\text{ and }P\left(H_{0}^{l}\right)=1-P\left(H_{1}^{l}\right) \end{array}$$

Then, the posterior probabilities $P\left(E_{C_{x_{i}}\left(l\right)}^{l}|H_{0}^{l}\right)$, $P\left(E_{C_{x_{i}}\left(l\right)}^{l}|H_{1}^{l}\right)$ are calculated by following equations,

(8) $$\begin{array}{l} P\left(E_{j}^{l}|H_{1}^{l}\right)=\frac{\left(s+\;c\left[j\right]\right)}{\left(s\times \left(k+1\right)+\sum_{i=0}^{k}c_{l}\left[i\right]\right)} \end{array}$$

(9) $$\begin{array}{l} P\left(E_{j}^{l}|H_{0}^{l}\right)=\frac{\left(s+\;c^{\prime }\left[j\right]\right)}{\left(s\times \left(k+1\right)+\sum_{i=0}^{k}c_{l}{}^{\prime }\left[i\right]\right)}\\ \;l=1,2,\ldots ,q,j=1,2,\ldots ,k \end{array}$$

where *s* is the smooth factor. *c*_*l*_[*i*] is the number of instances which just has *i* neighbors with *l*th disease in their *k* nearest neighbors; ${c^{\prime }}_{j}[i]$ is the number of instances which just has *i* neighbors without *l*th disease in their *k* nearest neighbors (Zhang and Zhou, 2007).

#### Cross-Validation

We used 5-fold stratified cross-validation with 1,000 repeats to avoid arbitrariness.

#### Ensemble Learning Method

In this paper, an ensemble learning method was designed to combine various features and develop high-accuracy prediction models (Lee and Soo, 2013; Yang et al., 2014; Wen et al., 2015). Previous studies have shown that combining predictions from different methods could achieve better and more robust results than using one algorithm alone. In this study, an ensemble classifier was generated using the linear weighted sum of outputs from classifiers based on four features.

Given *m* features, we build m individual feature-based MLKNN models, and use them as base predictors. Since features may make different contributes, it is natural to adopt weighted scoring ensemble strategy, which assigns *m* base predictors with m weights {*w*_1_, *w*_2_, …, *w*_*m*_}. For a testing instance, the *i*th predictor will give scores for *q* diseases, denoted as ${S_{i}=\left\{s_{i}^{1},s_{i}^{2},\ldots ,s_{i}^{q}\right\},\;i=1,2,\ldots ,m}_{.}$ The final prediction produced by the ensemble model is the linear weighted sum of outputs from base predictors.

(10) $$\begin{array}{l} \text{Ensemble Score}=\;\left[w_{1},w_{2},{\ldots ,w}_{m}\right]\times \left[\begin{array}{c} S_{1}\\ \begin{array}{c} S_{2}\\ \ldots \\ S_{m} \end{array} \end{array}\right] \end{array}$$

(11) $$\begin{array}{l} =\left[w_{1},w_{2},{\ldots ,w}_{m}\right]\times \left[\begin{array}{c} S_{1}^{1}\cdots S_{1}^{2}S_{1}^{q}\\ \vdots \ddots \vdots \\ S_{m}^{1}S_{m}^{2}\cdots S_{m}^{q} \end{array}\right] \end{array}$$

Tuning weights for base predictors are critical for the ensemble models. The weights are non-negative real values between 0 and 1, and the sum of weights equals 1. We adopt the internal 5-CV AUPR on training data is used as the fitness score (Lee and Soo, 2013; Yang et al., 2014; Wen et al., 2015).

#### Performance Evaluation

In the agent-activities prediction, the predicted scores for activities were usually merged for evaluation, and the metrics for ordinary binary classification were often adopted. The area under ROC curve (AUC) and the area under the precision-recall curve (AUPR) can be used to evaluate models regardless of any threshold. However, there are much more negative labels than positive labels in the agent-activities prediction, and machine-learning methods are likely to produce overestimated AUC scores. Since AUPR takes into account recall as well as precision, it is used as the most important metric.

We used the following evaluation metrics to evaluate the performance of machine-learning models: Precision, Accuracy (ACC), Recall, Specificity, Mathew's correlation coefficient (MCC) (12–16). These metrics can be calculated by the number of true positives (TP), false positives (FP), true negatives (TN), and false negatives (FN).

(12) $$\begin{array}{l} Precision=\frac{TP}{TP+FP} \end{array}$$

(13) $$\begin{array}{l} ACC=\frac{TP+TN}{TP+FN+TN+FP} \end{array}$$

(14) $$\begin{array}{l} Recall=\frac{TP}{TP+FN} \end{array}$$

(15) $$\begin{array}{l} Specificity=\frac{TN}{TN+FP} \end{array}$$

(16) $$\begin{array}{l} MCC=\frac{TP\times TN-FP\times FN}{\sqrt{\left(TP+FP\right)\times \left(TP+FN\right)\times \left(TN+FP\right)\times \left(TN+FN\right)}} \end{array}$$

Several metrics were designed for multi-label classification, i.e., Hamming loss, one-error, coverage, ranking loss and average precision. Hamming loss is the fraction of the wrong labels to the total number of labels. The one-error evaluates the fraction of examples whose top-ranked label is not in the relevant label set. The coverage evaluates how many steps are needed, on average, to move down the ranked label list so as to cover all the relevant labels of the example. The average precision evaluates whether the average fraction of relevant labels ranked higher than a particular label. Therefore, we adopt AUPR, average precision, one-error, coverage, ranking loss and hamming loss for the agent-activities prediction.

### Cytotoxicity Assays

#### Cell Culture and Reagents

K562 cells were purchased from Shanghai Cell Bank, Chinese Academy of Sciences. Cells were cultured in RPMI-1640 (Procell, China) with 10% FBS (Biowest, France) and 1% penicillin/streptomycin (Procell, China) at 37°C, in 5% CO_2_ humidified atmospheric air. All agents were purchased from TargetMol and dissolved in dimethyl sulfoxide (DMSO).

#### Cytotoxicity Assays

The effects of agents on K562 were determined using CellTiter-Glo® Luminescent Cell Viability Assay (Promega). Cells were seeded in 96-well plate at a density of 2 × 10^3^ cells/well and treated with different agents for 72 h together. An equal volume of CellTiter-Glo reagents was added to the cells in 96-well plates and mixed for 2 min on an orbital shaker and incubated for a further 10 min at room temperature. The luminescence of each well was measured by FlexStation3(Molecular Devices). The IC50 values were calculated using Graphpad Prism software. All experiments were performed in triplicate.

### Web Server Implementation

Systems Chemical Genetics-Drug (SCG-Drug, http://zhanglab.hzau.edu.cn/scgdrug) was built in Java, JavaScript, and Bootstrap with MySQL as the primary data store. The site is served with nginx on a server running CentOS 7.2. Two modules are used: the search module and the prediction module. The search module was implemented by an entry-name matching algorithm. By using this module, the server will return a list of partially matched terms and shows them in the dropdowns when users type only the starting characters of a gene, disease or drug in the search field. In the prediction module, there are two steps: data preprocessing and drug indication prediction. In the data preprocessing step, a Python script was used to produce the parameters matrix. In the drug indication prediction step, an R script was used to generate the result by calling the prediction model.

### Code and Data Availability

The R and Python scripts used to process the data and conduct the analyses described herein are available upon request. All of the intermediate data are available from the authors by request.

## Data Availability

Publicly available datasets were analyzed in this study. This data can be found here: http://zhanglab.hzau.edu.cn/scgdrug.

## Author's Note

Finding novel drugs or new uses for old drugs is a costly process. Previous studies have shown that genetics, which is best dedicated to revealing gene-disease links, makes great contributions to the pharmaceutical industry. On the other hand, most diseases are caused by multiple pathogenic factors. In this paper, we proposed that aiming at multiple genes associated with certain diseases rather than a single pathogenic factor is more efficient in identifying potential drugs. In addition, our results demonstrated the therapeutic potential of agents can be enhanced with the consolidation of genetic links between targets and diseases. In other words, simultaneously increasing the quantity and quality of target-disease associations can significantly increase the activity/druggability of agents. According to the above theories, we have established a drug-activity predictor with multi-label classification model based on the genetic information of drug targets (online service is freely available at SCG-Drug, http://zhanglab.hzau.edu.cn/scgdrug), which is of high value in prioritizing drug candidates.

## Author Contributions

H-YZ: conceptualization. YQ, Z-HL, and L-DZ: data curation. YQ, Z-HL, and Q-YY: formal analysis. H-YZ: funding acquisition. QZ, Z-JC, and XQ: investigation. H-YZ and L-DZ: methodology. JL and Y-ML: software. B-ML: conceived and designed the experiments. Y-HX: performed the experiments. H-YZ and L-DZ: supervision. H-YZ and L-DZ: validation. YQ, Z-HL, Q-YY, L-DZ, and JL: visualization. YQ, Z-HL, Q-YY, L-DZ, and H-YZ: writing–original draft. YQ, Z-HL, Q-YY, L-DZ, and H-YZ: writing–review and editing.

### Conflict of Interest Statement

H-YZ has received research/grant support from Wuhan Bio-Links Technology Co., Ltd. Huazhong Agricultural University and the developers of the methods for drug discovery and drug repositioning may benefit financially pursuant to the University's Policy on Inventions, Patents and Technology Transfer, even if these methods are not used in the commercialized therapy. The remaining authors declare that the research was conducted in the absence of any commercial or financial relationships that could be construed as a potential conflict of interest.
